# Biodegradation of decabromodiphenyl ether (BDE 209) by a newly isolated bacterium from an e-waste recycling area

**DOI:** 10.1186/s13568-018-0560-0

**Published:** 2018-02-24

**Authors:** Zhineng Wu, Miaomiao Xie, Yao Li, Guanghai Gao, Mark Bartlam, Yingying Wang

**Affiliations:** 1 Key Laboratory of Pollution Processes and Environmental Criteria (Ministry of Education), Tianjin Key Laboratory of Environmental Remediation and Pollution Control, College of Environmental Science and Engineering, Nankai University, Tianjin, 300350 China; 2 State Key Laboratory of Medicinal Chemical Biology, Nankai University, Tianjin, 300350 China; 3 College of Life Sciences, Nankai University, Tianjin, 300071 China

**Keywords:** *Stenotrophomonas*, Biodegradation, BDE 209, Genome sequencing, Real-time qPCR

## Abstract

Polybrominated diphenyl ethers (PBDEs) have become widespread environmental pollutants all over the world. A newly isolated bacterium from an e-waste recycling area, *Stenotrophomonas* sp. strain WZN-1, can degrade decabromodiphenyl ether (BDE 209) effectively under aerobic conditions. Orthogonal test results showed that the optimum conditions for BDE 209 biodegradation were pH 5, 25 °C, 0.5% salinity, 150 mL minimal salt medium volume. Under the optimized condition, strain WZN-1 could degrade 55.15% of 65 μg/L BDE 209 under aerobic condition within 30 day incubation. Moreover, BDE 209 degradation kinetics was fitted to a first-order kinetics model. The biodegradation mechanism of BDE 209 by strain WZN-1 were supposed to be three possible metabolic pathways: debromination, hydroxylation, and ring opening processes. Four BDE 209 degradation genes, including one hydrolase, one dioxygenase and two dehalogenases, were identified based on the complete genome sequencing of strain WZN-1. The real-time qPCR demonstrated that the expression level of four identified genes were significantly induced by BDE 209, and they played an important role in the degradation process. This study is the first to demonstrate that the newly isolated *Stenotrophomonas* strain has an efficient BDE 209 degradation ability and would provide new insights for the microbial degradation of PBDEs.

**Electronic supplementary material:**

The online version of this article (10.1186/s13568-018-0560-0) contains supplementary material, which is available to authorized users.

## Introduction

PBDEs are extensively used in various types of electronic equipment, furniture, plastics, textiles, etc. due to their excellent flame retardant performance (Leonel et al. 2014). Since PBDEs can be easily incorporated into different materials and products without making any covalent bonds, they could transport and migrate to different environment through volatilization and effusion etc. (Kim et al. 2007). Therefore, PBDEs have become widespread environmental pollutants and are ubiquitous in the environment (Birgul et al. 2012). Due to their persistency, long-range environmental transport potential and bioaccumulation ability, four PBDE congeners (i.e. tetrabromodiphenyl ether, pentabromodiphenyl ether, hexabromodiphenyl ether and heptabromodiphenyl ether) have been listed as new persistent organic pollutants (POPs) in 2009 (Stockholm Convention 2009). Previous studies demonstrated that PBDEs have a wide range of toxicities, including cytotoxicity (An et al. 2011), developmental neurotoxicity (Costa et al. 2014), genotoxicity (Ji et al. 2011), disruption of thyroid functions (Xu et al. 2014), reproductive toxicity (Muirhead et al. 2006) and carcinogenicity (Zhao et al. 2015). Penta-BDEs are reported to be the most toxic compounds among the PBDEs congeners. Deca-BDEs received the least concern among PBDEs congeners on account of its relatively lower toxicity (Gandhi et al. 2011). However, the higher-brominated PBDEs congeners (hepta-BDEs to deca-BDEs) could be degraded/transformed to lower-brominated PBDEs congeners which seems to be more toxic in various environments such as soil, aquatic ecosystems (Huang et al. 2014). Therefore, PBDEs had posed a great threat to both human health and the global environment.

PBDEs are lipophilic, and have very low solubility in water (ng/L to μg/L) (He et al. 2006). Hence, accumulation of PBDEs in the environment, as well as in fatty tissues of humans and other animals are very likely to happen. Therefore, increased number of research have focused on the distribution and level of PBDEs in different environments (Kim et al. 2014), biota (Vorkamp et al. 2011) and in humans (Abdallah and Harrad 2014) worldwide. In contrast, there are very few reports on the degradation of PBDEs, especially on the biodegradation of PBDE compounds. Given their worldwide distribution, it is important to develop effective degradation methods to remove PBDEs from environment, especially the most abundant congener BDE 209 (Ma et al. 2012; Mcgrath et al. 2017). The reported microbial degradation of PBDEs have focused on the low PBDE congeners. For instance, a previous study showed *Sphingomonas* sp. SS3 can transform and grow on 4-bromodiphenyl ether (Schmidt et al. 1993). Robrock et al. reported the bacterial transformation of mono- to hexa-BDEs at μg/L levels (Robrock et al. 2011). More recently, *Pseudomonas stutzeri*, *Pseudomonas putida* sp. strain TZ-1 and *Pseudomonas* sp. were used to biodegrade different low PBDE congeners (Xin et al. 2014; Yang et al. 2015; Zhang et al. 2013). To the best of our knowledge, little information on microbial degradation of BDE 209 is available under aerobic condition.

In the present study, we investigated the BDE 209 biodegradation by a newly isolated bacterium from an e-waste recycling area in Tianjin, China. This bacterial strain can utilize BDE 209 as its carbon source effectively under aerobic conditions. Orthogonal tests were designed to acquire optimal condition for biodegradation of BDE 209. A first-order kinetics model was used to fit the BDE 209 degradation kinetics. Moreover, the intermediate products were detected by GC–MS and UPLC–MS. Furthermore, key BDE 209 degradation genes were identified through the complete genome sequencing analysis as well as real-time qPCR tests.

## Materials and methods

### Chemicals

BDE 209 standard solution (concentration 50 µg/mL) in isooctane: toluene (9:1, v: v) and standard solutions containing 14 PBDE congeners (concentration 5 µg/mL, decabromodiphenyl ether was 25 µg/mL) in isooctane were purchased from AccuStandard (New Haven, CT, US). BDE 209 (99% purity) was bought from J&K Scientific Ltd and dissolved in tetrahydrofuran (THF) as stock solutions for bacteria isolation and biodegradation experiments. Isooctane was HPLC grade, and the other chemicals and solvents used in the experiments were analytical grade.

### Bacterial isolation

Soil samples (0–15 cm) were collected in November 2014 from Ziya e-waste recycling area in Tianjin, north China (38°52′33.1″N, 116°49′48.7″E). Bacterium capable of using BDE 209 as the carbon source was enriched and isolated. The minimal salt medium (MSM) contained basal mineral medium (BMM) (Na_2_HPO_4_·12H_2_O 25.6 g/L, KH_2_PO_4_ 3 g/L, (NH_4_)_2_SO_4_ 1.77 g/L), and trace mineral solution (1% v/v) containing HCl 20 mL/L, CaCO_3_ 8 g/L, FeCl_3_·6H_2_O 7.74 g/L, MnCl_2_·4H_2_O 1.15 g/L, CuSO_4_·5H_2_O 0.146 g/L, CoCl_2_·6H_2_O 0.13 g/L, ZnO 0.4 g/L, H_3_BO_3_ 0.124 g/L, EDTA Na_4_·2H_2_O 79.2 g/L, MgCl_2_·6H_2_O 13.42 g/L, Na_2_MoO_4_·2H_2_O 1.04 g/L. The pH of MSM was 7.4.

5 g soil sample was suspended in 50 mL sterilized saline solution (0.85%, w: v), and vortexed for 5 min, followed by 10 min standing at room temperature. The supernatant was passed through 0.45 μm sterile filters, and then 1 mL filtrate was transferred to sterilized MSM containing 10 mg/L BDE 209. The Erlenmeyer flasks (250 mL) were incubated 30 ± 1 °C, 150 rpm for 7 days. After three rounds of the supernatant transferring, which means transfer MSM with lower BDE 209 concentration to fresh MSM with higher BDE 209 concentration, the BDE 209 concentration finally reached 30 mg/L. Then, the enrichment culture was streaked in solid LB (Luria–Bertani) medium plates. A single colony on plates were picked and inoculated into LB medium for further identification and BDE 209 degradation. The isolated bacterium was designated as strain WZN-1.

### Bacterial identification

The isolated bacterium was identified based on 16S rRNA sequence analysis and morphological properties. The genomic DNA of strain WZN-1 was extracted by the UltraClean^®^ Microbial DNA Kit (MoBio, USA). The 16S rRNA gene sequence was amplified by PCR using the universal primers (Lane 1991). The PCR products were sent to the AuGCT DNA-SYN Biotechnology Co., Ltd. (Beijing, China) for sequencing. The sequence was aligned and compared with those in the GenBank database, using ClustalW with default parameter values. A phylogenetic tree was constructed by the neighbor-joining method using the program MEGA 6.0 software. The morphological characteristics were examined by scanning electron microscopy (SEM) using an S-3500 N SEM (Hitachi, Japan).

### Optimization of BDE 209 degradation conditions

Strain WZN-1 was grown in 250 mL Erlenmeyer flask containing 100 mL of LB medium and incubated at 35 ± 1 °C for 36 h until its late-exponential phase. Then harvested by centrifugation at 7000 rpm for 5 min, and washed with 0.85% sterilized saline solution for three times. The initial bacterial concentration was 2 × 10^4^ cells/mL. Orthogonal test of five factors with five levels was used to identify the optimal BDE 209 degradation condition (Xu et al. 2011). The selected five factors were pH, temperature, salinity, MSM volume and initial BDE 209 concentration, respectively. The different levels of the five selected factors were shown in Additional file 1: Table S1. A total of 25 experimental combinations were carried out (Additional file 1: Table S2). All the experiments were set in triplicate. The BDE 209 concentration was measured after 7 days incubation. The optimal BDE 209 degradation conditions were determined by analyzing the orthogonal experiment results.

### Biodegradation of BDE 209 by strain WZN-1 under the optimal condition

Batch biodegradation experiments were performed in 250 mL serum bottles under the optimized BDE 209 degradation condition, temperature 25 ± 1 °C, 0.5% salinity, 150 mL MSM volume. BDE 209 concentration was 65 μg/L. The pH was adjusted to 7 by HCl, because strain WZN-1 showed better growth at pH 7 than pH 5 according to the orthogonal test. The initial bacterial concentration was 6 × 10^4^ cells/mL. Un-inoculated control bottles were kept to account for the abiotic loss of BDE 209. Glassware used in this experiments were carbon free (Vital et al. 2012). All experiments were carried out in triplicate. Samples were taken at regular time intervals. Bacterial growth was monitored by FCM (flow cytometry) and BDE 209 concentration was determined by GC–MS. The FCM analysis was carried out on a CyFlow Space instrument (Partec, Hamburg, Germany). Detailed procedure was as previously described (Hammes et al. 2008; Prest et al. 2013; Wang et al. 2009). The specific instrumental gain settings were as follows: green fluorescence = 250, red fluorescence = 700, SSC = 250, FSC = 700. The same setting was used for all samples tested in this study.

The first-order kinetics model was used to analyze BDE 209 biodegradation data by strain WZN-1 (Eq. 1) (Peng et al. 2015).

1 $$ \frac{{C_{t} }}{{C_{0} }} = e^{ - kt} $$

where *C*_0_ is the initial BDE 209 concentrations, *C*_t_ refer to BDE209 concentration at time *t*; *k* and *t* are the degradation rate constant and degradation time, respectively.

The half-lives (*T*_1/2_) of BDE 209 biodegradation were determined by the algorithm (Eq. 2) (Peng et al. 2015).

2 $$ T_{1/2} = \ln 2/k $$

### Intermediate products extraction and analysis

Samples containing PBDEs were extracted by liquid–liquid extraction (Lee and He 2010). The extraction efficiencies of different PBDEs congeners, measured as the amount of PBDEs mixture recovered compared to the amount of PBDEs added to the control samples, with an extraction variation range of 83.09–103.21%. The BDE 209 concentration and the 13 lower PBDEs congeners were identified and quantified by GC–MS (7890A-5975C, Agilent Technology, USA) equipped with Rtx-1614 GC column (15 m × 0.25 mm × 0.10 μm). Automatic splitless injection was used with 1 μL injection volume at 280 °C. The carrier gas was helium (flow rate 2 mL/min). Pressure was kept constant at 21.77 psi. The oven temperature was started at 110 °C for 3 min, and then was heated to 200 °C at a rate of 25 °C/min, 15 °C/min up to 280 °C, and finally 20 °C/min increased to 305 °C held for 5 min. The total program time was 18.183 min. The ion source temperature was set at 150 °C, and the auxiliary temperature was 300 °C. Negative chemical ionization (NCI) mode was used in mass spectrometry, reagent gas was methane, and the ionization energy was 70 eV. The detection was selected ion monitoring in the NCI mode. The *m/z* 486.7 and 488.7 were monitored ions for BDE 209, and ions *m/z* 79 and 81 were selected for the 13 lower PBDEs congeners except BDE 209.

1 mL of the extracted top organic layer (method is the same with BDE 209 extraction) was transferred to dry test tubes, dried under nitrogen, then dissolved in 1 mL of methanol, and filtered through a 0.22 μm membrane filter. The UPLC–ESI–MS method was developed for the determination of OH-PBDEs (Wang et al. 2011). UPLC–MS (Waters ACQUITY UPLC H-CLASS, Waters, Milford, MA, USA) equipped with an ESI source (Waters, Milford, MA, USA) was used to identify intermediate products. The parameters were as follows: capillary voltage, 10 kV; source temperature, 160 °C; desolvation temperature, 400 °C. The mass spectrometer was operated in negative electrospray ionization (ESI-) mode using methanol–water (v: v, 90/10) as the mobile phase with a flow rate of 2 mL/min.

### Complete genome sequencing and degradation genes identification

The genomic DNA of strain WZN-1 was extracted as described above in “Bacterial identification” section. The complete genome sequencing of WZN-1 was finished using Illumina Hiseq 4000 and PacBio RSII sequencing platform. The detailed sequencing information were described in a previous study (Wu et al. 2017). The specific degradation genes were identified and searched from the annotation of all the genes in WZN-1 use key words (such as dehalo, ring-opening) for further real-time qPCR analysis.

### Real-time qPCR

Strain WZN-1 was incubated in MSM medium supplemented with 65 μg/L BDE 209 under the optimized BDE 209 degradation conditions (same with “Biodegradation of BDE 209 by strain WZN-1 under the optimal condition batch” section). Strain WZN-1 was grown in MSM medium without BDE 209 was set as the control. The initial bacterial concentration was 1.36 × 10^6^ cells/mL so that the biomass can meet the need for RNA extraction. Samples were collected in 5, 10, and 20 days from the bottles for the FCM analysis and real-time qPCR analysis. The total RNA of the 10 mL liquid samples (10^7^ cells/mL) were extracted using the Total RNA Purification Kit (GeneMark, Taichung, Taiwan) as described by the manufacturer. Genomic DNA was eliminated by RNase-free DNase I treatment during the extraction procedure. The SAMreal™ One-Step RT qPCR Kit (SYBR Green with low ROX) (GeneMark, Taichung, Taiwan) was used for the real time qPCR experiments in 96-well plates performed on the LightCycler^®^ 96 real-time PCR system (Roche, Switzerland). The primers for real-time PCR were designed with the Primer Premier 5.0 software and synthesized by Sangon Biotech Co (Shanghai, China) (Table 1). The 16S rRNA gene was used as a reference gene to normalize gene expression in strain WZN-1 (Wang and Shao 2012). Each real-time qPCR mixture (20 µL) contained 1 μL RNA, 1 μL enzyme mix, 1 0 μL 2× RT qPCR reaction (low ROX), 0.8 μL primers and 7.2 μL Rnase-free water. For each qPCR experiment, control reactions without reverse transcriptase were conducted to verify the absence of genomic DNA. Cycling parameters were designed as follows: reverse transcription at 50 °C for 3 min, initial denaturation at 95 °C for 10 min, followed by 40 cycles of 95 °C for 5 s and 60 °C for 30 s. The fluorescence values were measured during each annealing step. The melting curve was used to verify the specificity of the real-time qPCR reaction. The relative fold change in mRNA quantity was calculated for the gene of interest in each sample using the 2^−ΔΔCt^ method (Livak and Schmittgen 2001). All experiments were carried out in triplicate.

**Table 1:** Genes and corresponding primers for the real-time qPCR

Gene name	Gene ID	Gene function	Forward (5′ to 3′)	Reverse (5′ to 3′)	References
16S rRNA	–	Reference gene	CGGTGAATACGTTCYCGG	GGWTACCTTGTTACGACTT	Wang et al. (2015)
Hydrolase	CCR98_00905	Alpha/beta hydrolase	AGCTATTACTGGCGCACGTT	TGTACTCGTAGCGGCTGTCA	Wu et al. (2017)
Dioxygenase	CCR98_02495	Aromatic ring-opening dioxygenase	AAGGCCGAGCAGGATTATCT	GACAGCGTCATGCTCTTCAC	Wu et al. (2017)
Dehalogenase gene 1	CCR98_02005	Haloacid dehalogenase	ATCTGTTCGCCTCGCTGAT	ATAGACCGAAATGCCAGCAC	Wu et al. (2017)
Dehalogenase gene 2	CCR98_19135	Haloacid dehalogenase	CAGCATCACCCACAACCTG	CTGCTCTACCCACTCGATGAA	Wu et al. (2017)

### Strain accession number

The 16S rRNA sequence of strain WZN-1 was deposited in NCBI GeneBank with the accession number of KY000522. The complete genome sequence of strain WZN-1 have been deposited in NCBI with a GenBank accession number of CP021768.

## Results

### Identification of strain WZN-1

A pure strain that can utilize BDE 209 as its carbon source was isolated from the mixed culture after 1 month of enrichment. This strain was designated as WZN-1. After incubation at 37 °C for 2 days, the colonies of strain WZN-1 on LB medium agar plates were approximately 1–2 mm in diameter. Cells of strain WZN-1 were rod-shaped, and measured 0.25–0.7 μm by 0.3–2 μm (Additional file 1: Fig. S1a). This strain was a Gram-negative bacterium. The physiological and biochemical characteristics of strain WZN-1 are shown in Additional file 1: Table S3. According to a BLAST search against the NCBI GenBank, the 16S rRNA sequence of strain WZN-1 has a high sequence similarity with *Stenotrophomonas* (Additional file 1: Fig. S2). The taxonomic position showed that the strain WZN-1 was a member of the *Stenotrophomonas* subgroup in the class γ-proteobacteria. The collection number of *Stenotrophomonas* sp. strain WZN-1 was CGMCC No. 12918 in China General Microbiological Culture Collection Center. Furthermore, this strain exhibited growth properties as a facultative oligotroph. The strain exhibited good growth at a wide range of carbon concentrations (13–13,000 mg C/L) (Additional file 1: Fig. S3). Compared with *Escherichia coli* BL21, strain WZN-1 demonstrated obvious growth advantages under oligotrophic condition. According to the un-published of our group, the yield a common copiotrophic strain *Escherichia coli* BL21 growing with 13 mg C/L was 8 × 10^6^ cells/mL (data not shown), which is 100 times lower than that of strain WZN-1.

### Optimization of BDE209 degradation conditions

Orthogonal tests were designed to acquire optimal condition for biodegradation of BDE 209 (Additional file 1: Table S1). The results of the orthogonal tests are presented in Additional file 1: Table S2. Range analysis and variance analysis of the orthogonal test were carried out to evaluate the influence of different factors on BDE 209 biodegradation (Additional file 1: Tables S2, S4). The optimum biodegradation conditions were pH 5, 25 °C, 0.5% salinity, 150 mL MSM volume. The results showed that pH was the most important factor (*R* = 49.41), followed by MSM volume (*R* = 15.13), salinity (*R* = 13.49), temperature (*R* = 10.33) and BDE 209 concentration (*R* = 6.51) (Additional file 1: Table S2). The variance analysis demonstrated that the most significant factor for BDE 209 degradation was pH (*p* = 0.01), followed by salinity (*p* = 0.40), volume (*p* = 0.44), temperature (*p* = 0.67) and BDE 209 concentration (*p* = 0.91) (Additional file 1: Table S4).

### Biodegradation of BDE 209 and kinetic analysis

**Fig. 1:**
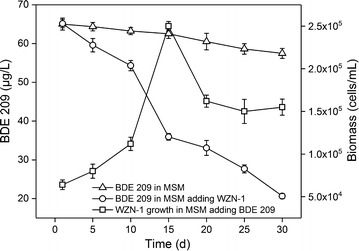
Biodegradation of BDE209 by strain WZN-1 under the optimized condition. The error bars represent standard deviation of triplicate measurements

**Fig. 2:**
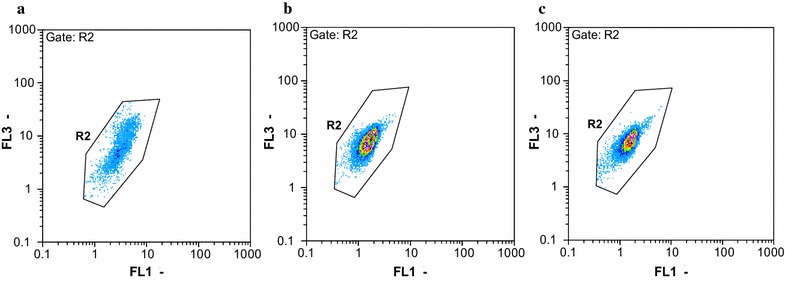
Examples of flow cytometric dot plots of strain WZN-1 at different time during BDE 209 degradation: **a** 0 day; **b** 15 days; **c** 30 days. FL1 represents green fluorescence was collected at FL1 = 520 ± 20 nm, and FL3 represents the red fluorescence was collected at FL3 = 630 nm

The BDE 209 biodegradation by the strain WZN-1, as well as the bacterial growth were investigated simultaneously in MSM under the optimal condition, to which 65 μg/L BDE 209 was applied as carbon and energy source. The results showed strain WZN-1 could biodegrade 55.15% of the initial BDE 209 in the batch experiments over a 30-d incubation period (Fig. 1), and the pH values remained at initial levels. The biomass of strain WZN-1 rapidly increased with time in the first 15 days incubation, and reached the maximum concentration of 2.50 × 10^5^ cells/mL, then decreased from 15 to 25 days and kept relatively stable from 25 to 30 days. Figure 2 shows the flow cytometric dot plots of strain WZN-1 growth measured during the biodegradation process. The results indicated that the cellular properties (e.g. cell size and cellular nucleic acid content) of strain WZN-1 was consistent with the BDE 209 degradation rates. During the first 15 days, BDE 209 biodegradation efficiencies were higher concomitant with the rapid bacteria growth. After that, the strain gradually adapted to the BDE 209 environment, so that the BDE 209 degradation rate maintained a stable increase from 15 to 30 days, which means that the strain has a strong BDE 209 biodegradation capacity (Guangyu et al. 2013). The kinetic analysis indicated that the biodegradation of BDE 209 by strain WZN-1 well fitted the first-order kinetics (Additional file 1: Fig. S4). BDE 209 degradation rate constant was 0.0456 ± 0.0095/days, the coefficient of determination *R*^2^ was 0.7862, and the half-lives was 15.8869 ± 4.6807 days.

### Identification of intermediate products and biodegradation pathway

**Fig. 3:**
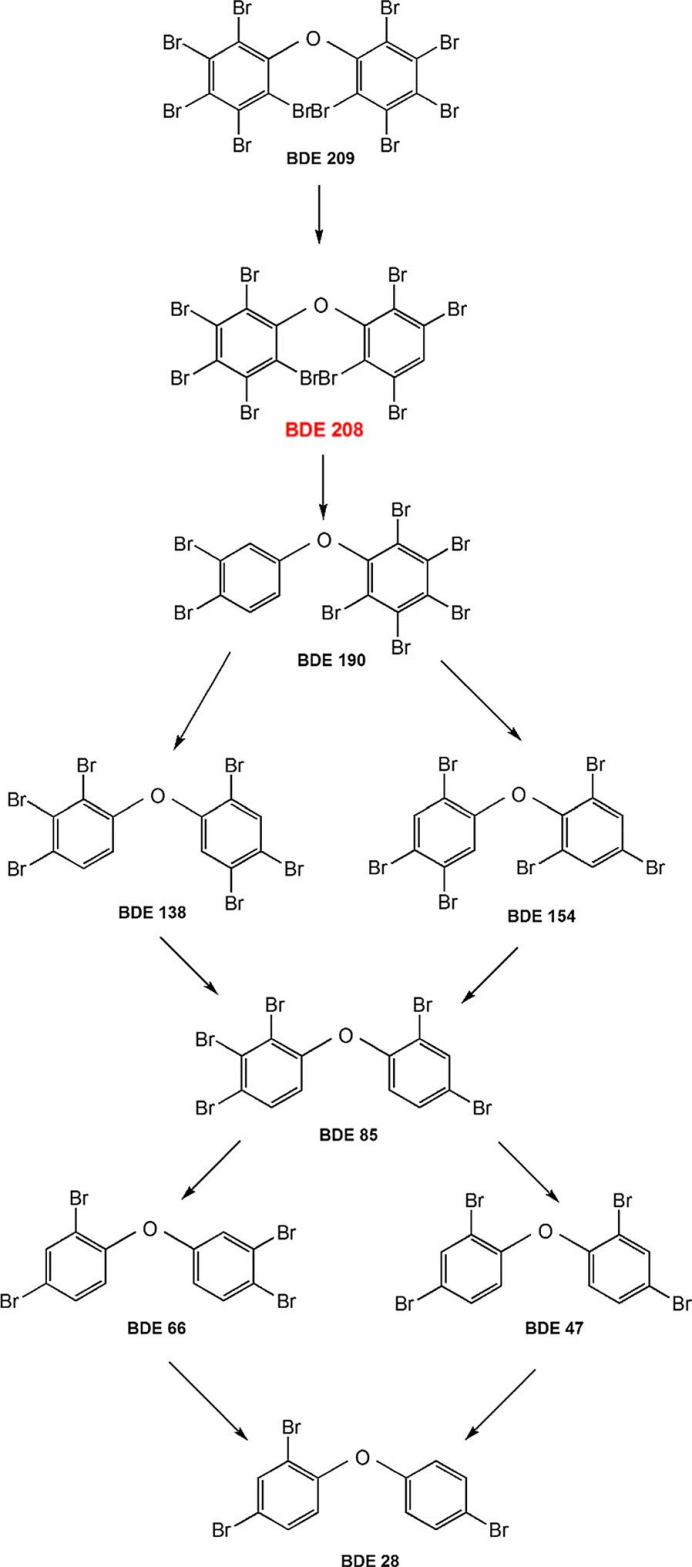
A proposed debromination pathway for BDE 209 biodegradation by strain WZN-1

**Fig. 4:**
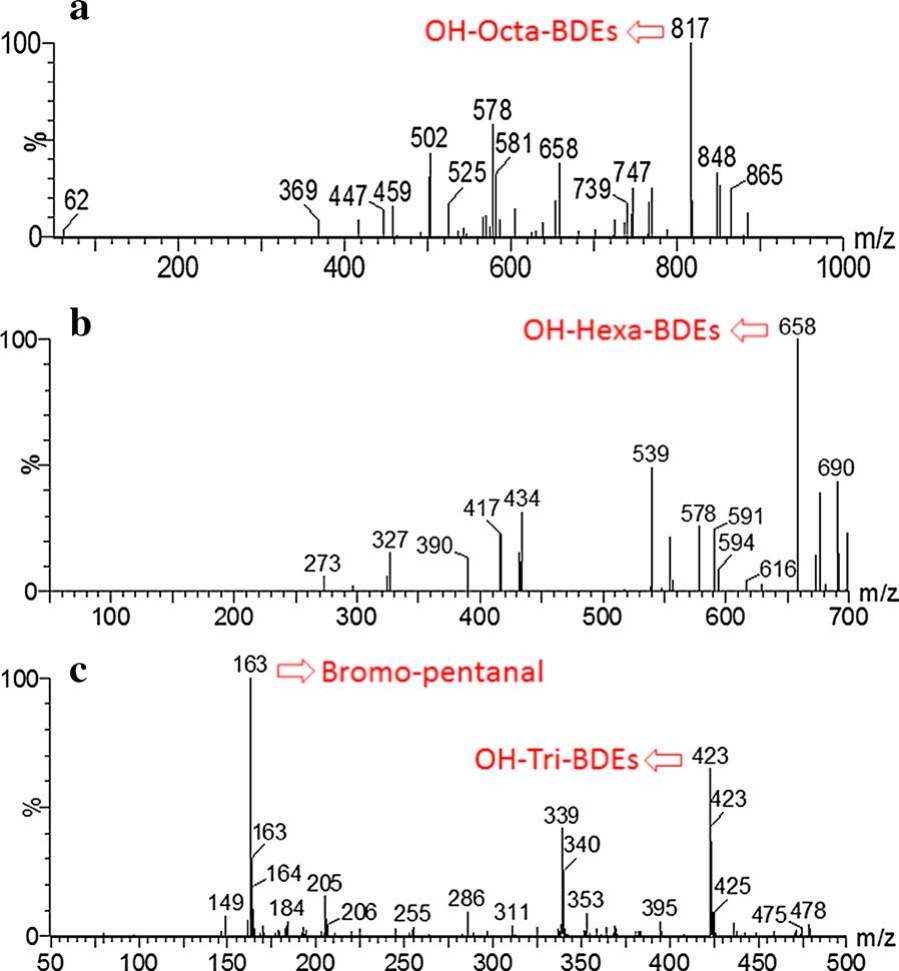
Possible intermediate products identified by UPLC–MS during BDE 209 biodegradation by strain WZN-1 under the optimal condition: **a** OH-Octa-BDEs (*m/z* 817); **b** OH-Hexa-BDEs (*m/z* 658); **c** OH-Tri-BDEs (*m/z* 423) and bromo-pentanal (*m/z* 163)

To identify the intermediate products and biodegradation pathway of BDE 209 by strain WZN-1, the BDE 209 biodegradation products in different times were detected and identified by GC–MS (Additional file 1: Fig. S5). The proposed debromination pathway for BDE 209 biodegradation by WZN-1 strain is shown in Fig. 3. The GC–MS analysis confirmed that one nona-BDEs (BDE 208), one hepta-BDE (BDE 190), two hexa-BDEs (BDE 138 and BDE 154), one penta-BDE (BDE 85), two tetra-BDEs (BDE 47 and BDE 66), and one tri-BDE (BDE 28) were formed during BDE 209 degradation by strain WZN-1. This indicated that BDE 209 was first converted by para debromination to BDE 208 (Guangyu et al. 2013). Then, the BDE 208 was debrominated to BDE 190, and BDE 190 was converted by ortho and meta debromination to BDE 138 and BDE 154. After that, BDE 138 and BDE 154 were converted to BDE 85 by ortho and meta debromination. BDE 66 and BDE 47 were formed by the ortho and meta debromination of BDE 85. BDE 66 was finally converted to BDE 28 by meta debromination, and BDE 47 converted to BDE 28 by ortho debromination. In comparison, there was no detectable change of lower PBDEs congeners occurred over time in the control experiments (Additional file 1: Fig. S5). Furthermore, samples were full-scanned by UPLC–MS and the mass spectra were recorded in full scan mode (*m/z* 50–1000). The proposed OH-PBDEs and open-ring products based on UPLC–MS analysis were listed in Fig. 4. Four major biodegradation products were deducted, including three OH-PBDEs: OH-Octa-BDEs (*m/z* 817), OH-Hexa-BDEs (*m/z* 658), OH-Tri-PBDEs (*m/z* 423); and one possible ring opening product bromo-pentenal (*m/z* 163).

### Genome analysis of strain WZN-1 and real-time qPCR

**Fig. 5:**
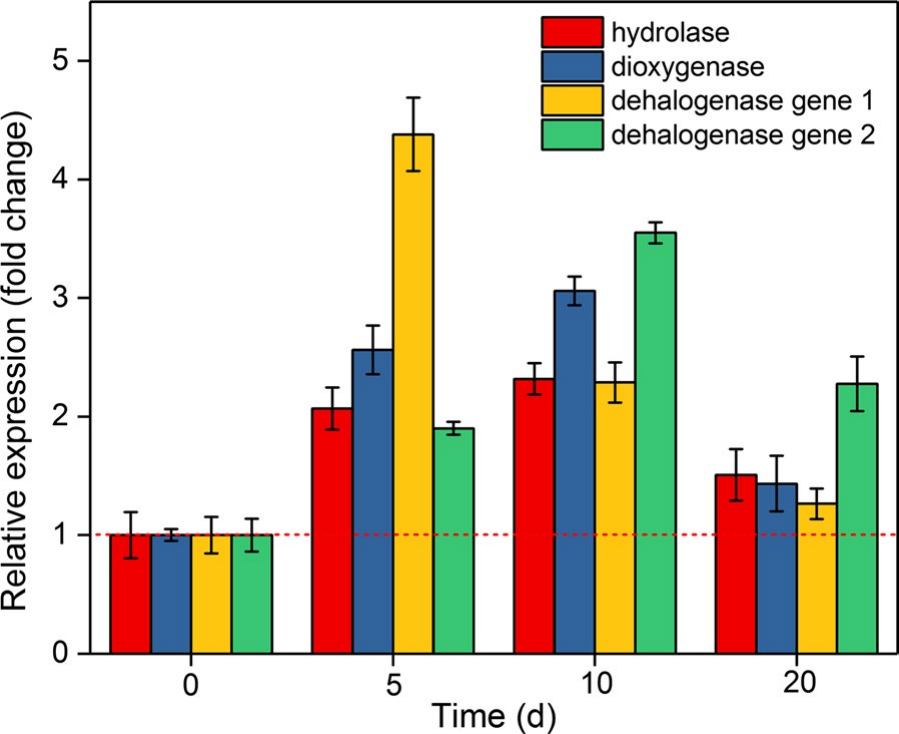
The relative expression of four BDE 209 degradation genes during the degradation process

According to the genome sequencing, the genome size of strain WZN-1 is 4,512,703 bp with a GC content of 66.62%. The genome contained 4137 genes, the total length of genes was 3,961,347 bp, which makes up 87.78% of genome (Wu et al. 2017). Based on the functional annotation of the 4137 genes, four genes were identified and supposed to have BDE 209 degradation ability (Table 1). In this study, the real-time qPCR analysis showed that the expression of the four identified BDE 209 degradation genes was significantly induced and upregulated by BDE 209, demonstrating their involvement in BDE 209 degradation process (Fig. 5). The expression levels of the four genes were showed similar trend, where the level first increased and then decreased along the degradation process. This is consistent with the strain WZN-1 growth in BDE 209, which is rapidly increased in the first 5 days incubation, and reached the maximum concentration of 1.60 × 10^7^ cells/mL, then decreased to 8.76 × 10^6^ and 2.31 × 10^6^ cells/mL on 10 and 20 days, respectively. Generally, the expression levels of dehalogenase gene 1 and dehalogenase gene 2 were significant higher than the hydrolase gene and dioxygenase gene. The dehalogenase gene 1 showed the highest relative expression (4.38-fold) on 5 days, decreased to 2.28-fold on 10 days, and back to the initial level on 20 days. However, the expression of the dehalogenase gene 2, hydrolase gene and dioxygenase gene were raised in the first 10 days, and showed the highest expression levels on 10 days, but their expressions were dropped on 20 days. The statistical analysis showed that all the three genes showed a significant expression level (*p* < 0.05) on 5 and 10 days. The expression of the four genes in strain WZN-1 enabled this strain to deplete BDE 209.

## Discussion

PBDEs are persistent in various environments, and tend to accumulate in the biota (Mackintosh et al. 2015). Despite the adverse environmental impact of decabromodiphenyl ether (BDE 209), very limited information is available for its microbial degradation under aerobic conditions. The present study reported the aerobic biodegradation of BDE 209 by a newly isolated strain, *Stenotrophomonas* sp. strain WZN-1. In previous studies, *Stenotrophomonas* spp. has been found to be ubiquitous in marine, soil, rhizosphere of diverse plants (Denton and Kerr 1998) and polluted environments (Dungan et al. 2003). The genus *Stenotrophomonas* has been recorded as a promising candidate for biotechnological applications involved in the detoxification of various man-made pollutants because its broad spectrum of metabolic properties (Ryan et al. 2009), including utilization of polycyclic aromatic hydrocarbons (Juhasz et al. 2000), benzene, toluene, ethylbenzene (Lee et al. 2002), chlorinated compounds (Somaraja et al. 2013), heavy metals (Ghosh and Saha 2013) and xenobiotics (Fuller et al. 2010). Therefore, *Stenotrophomonas* spp. has a great application value in the field of environmental remediation.

According to the orthogonal experiment results, pH value is a vital factor influencing the bacterial biodegradation ability of various organic pollutants. pH could adjust the metabolism and growth of the microorganisms via controlling the enzymatic activities (Jin et al. 2012). The present study showed that strain WZN-1 preferred acidic environment at pH 5, where its BDE 209 degradation rate was significantly higher than those at other pH levels (Additional file 1: Table S2). Comparing to the effect of pH, the influence of temperature on the BDE 209 biodegradation by WZN-1 was not significant. It is consistent with a previous study, with the increasing temperature from 20 to 40 °C, the BDE 209 degradation rate ranged from 17.83 to 28.15%; the highest degradation rate was observed at 25 °C (Xin et al. 2014). The MSM salinity affects the osmotic pressure, so that will affect the growth of bacteria and its degradation rate on BDE 209. Our results indicated that strain WZN-1 had considerate tolerance for NaCl over the range from 0 to 4%. The highest BDE 209 degradation rate of 29.47% occurred at 0.5% salinity. The volume of the MSM also apparently influences BDE 209 degradation rate by WZN-1 strain. With the volume increases from 50 mL to 250 mL, the BDE 209 degradation rate ranged from 16.14 to 31.18%, and the highest degradation rate was observed at 150 mL, which indicated that the oxygen plays a crucial role in the BDE 209 biodegradation system. Comparing to other factors tested, the initial concentrations of BDE 209 had no significant impact (*p* = 0.91) on the BDE 209 biodegradation efficiency by strain WZN-1.

Previously, researches on the biodegradation of BDE 209 and some other PBDEs congeners have been reported (Table 2). A number of bacteria including *Pseudomonas* spp., *Sphingomonas* spp., *Lysinibacillus fusiformis* DB-1, *Bacillus cereus* JP12, and *Phlebia lindtneri* JN45 have been demonstrated to transform and degrade different PBDE congeners (Kim et al. 2007; Lu et al. 2013; Xu and Wang 2014). A few microbial degradations of BDE 209 have also been reported. For instance, it was shown the *Bacillus cereus* JP12 could degrade over 88% of BDE 209 (initial concentration 1 mg/L) after 12 days incubation (Lu et al. 2013). Besides, 1 g/L bacterial cells of *Enterococcus casseliflavus* could degrade 52.2% of 1 mg/L BDE 209 in 7 days (Tang et al. 2016). The present study showed strain WZN-1 could biodegrade 55.15% of 65 μg/L BDE 209 over a 30-day incubation period under the optimized aerobic degradation conditions. Besides, strain WZN-1 exhibited high viability and was still culturable when exposed to 5 mg/L BDE 209 in MSM (data not shown). The biodegradation efficiency of BDE 209 by strain WZN-1 was comparable to the previous reports that microbial degradation of BDE 209 at μg/L to mg/L level (Table 2). The degradation ability of strain WZN-1 presented in this study could provide new options for the remediation of PBDEs.

**Table 2:** Comparison of the PBDEs degradation efficiency reported in previous reports

PBDEs	Time	Initial concentration	Degradation bacteria	Degradation efficiency	References
BDE 209	30 days	65 μg/L	*Stenotrophomonas* sp. strain WZN-1	55.15%	This study
Diphenyl ether, 4-bromo-, 2,4-dibromo-,4,4′-dibromodiphenyl ether	8 days	1 g/L diphenyl ether	*Sphingomonas* sp. PH-07	100% (diphenyl ether), 23% (4-bromo), 14% (2,4-dibromophenyl ether), 8% (4,4′dibromodiphenyl ether)	Kim et al. (2007)
Tetra-, penta- and hexa-BDEs	3 days	17 ng/mL	*Rhodococcus jostii* RHA1, *Burkholderia xenovorans* LB400, *Pseudonocardia dioxanivorans* CB1190	RHA1 transformed greater than 90% of mono- and di-BDE congeners, LB400 transformed 10–45% of penta-BDE congeners	Robrock et al. (2009)
Octa-BDE	2 months	45 nM nona-BDE, 181 nM octa-BDEs, 294 nM hepta-BDE, and 19 nM hexa-BDE	*Dehalococcoides* species	–	Lee and He (2010)
BDE 47, 99, 100	14 days	1180 nM	*Dehalococcoides* and *Desulfovibrio* spp.	88–100%	Lee et al. (2011)
BDE 209	6 days	6 mg/L	*Lysinibacillus fusiformis* DB-1	< 20%	Deng et al. (2011)
BDE 209	90 days	10 μM of BDE 209	*Pseudomonas* spp.	< 12%	Qiu et al. (2012)
BDE 209	12 days	1 mg/L	*Bacillus cereus* JP12	88%	Lu et al. (2013)
Octa and penta-BDE technical mixtures	21 weeks	280 nM PBDEs in an octa-BDE mixture	*Acetobacterium* sp. strain AG *Dehalococcoides* sp. strain DG	96%	Ding et al. (2013)
BDE 47	2 weeks	20 μg/L	*Pseudomonas stutzeri*	97.94%	Zhang et al. (2013)
BDE 47	7 days	50 μg/L	*Pseudomonas putida* sp. strain TZ-1	49.96%	Xin et al. (2014)
BDE 209	7 days	1 mg/L	*Enterococcus casseliflavus*	52.2%	Tang et al. (2016)
BDE 209	12 days	0.5 mg/L	*Staphylococcus haemolyticus* LY1 and *Bacillus pumilus* LY2	29.8 and 39.2%	Wang et al. (2016)
BDE 209 BDE 209	144 h 5 days	50 mg/L 20 mg/L	*Rhodococcus* sp. *Pseudomonas aeruginosa*	65.1% 85.12%	Liu et al. ( 2015, 2016)

–: not mention

Additionally, biodegradation of BDE 209 by strain WZN-1 was first-order kinetics. The half-life of BDE 209 was 15.8869 ± 4.6807 days, which revealed that BDE 209 could be rapidly degraded by strain WZN-1. PBDEs have very low water solubility and high octanol–water partition coefficient, hence they exhibited low biodegradation in different environment (Stiborova et al. 2015). The microbial degradation kinetics of different PBDEs congeners in sediment and in sewage sludge have been investigated. For instance, Huang and colleagues found the anaerobic debromination rate constant for BDE 209 was 0.022/days, the half-life was 31.5 days in sediment (Huang et al. 2014). Another study also investigated the degradation of PBDEs in sediment (Yang et al. 2015). Their results showed that the degradation rate constants and half-lives were 0.347/days and 2 days, 0.128/days and 5.4 days, 0.056/days and 12.4 days, 0.050/days and 13.9 days, 0.047/days and 14.8 days for BDE 15, BDE 28, BDE 47, BDE 99 and BDE 100, respectively. Microbial degradation of BDE 209 in sewage sludge was much slower than that in sediment. It was reported that the degradation constant ranged between 0.00277 and 0.00379/days in sewage sludge under aerobic conditions (Stiborova et al. 2015). So far, there are only few studies described the biodegradation kinetics of BDE 209 in water. Comparing to previous studies, strain WZN-1 showed more effective biodegradation of BDE 209 in MSM with degradation rate constants 0.0456 ± 0.0095/days and half-life 15.8869 ± 4.6807 days.

As mentioned in previous studies, enzymatic dehalogenation involves the removal of halogen atoms by hydrolytic, reductive, or oxygen dependent dehalogenation (Reineke 2001). Based on the observed lower PBDEs congeners, one of the major biodegradation mechanisms of BDE 209 by WZN-1 strain was debromination (Fig. 3, Additional file 1: Fig. S5). However, no bromide ions were detected by ion chromatography during BDE 209 degradation, which may due to the relatively low concentrations of bromide released, or the concentration of bromide ions is lower than the detection limit of the instrument. To date, information on the aerobic degradation of BDE 209 was relatively limited. It was reported that deca-BDE could be aerobically debrominated to octa-BDE and hepta-BDE congeners (Deng et al. 2011). In addition, lower brominated PBDEs such as BDE 196, BDE 197, BDE 202, BDE 203, and BDE 183 were detected during BDE 209 biodegradation (Guangyu et al. 2013). The ring of fully brominated BDE 209 is difficult to open directly because of its symmetric molecular structure and enhanced chemical stability by higher bromine constitution. Therefore, the biodegradation of BDE 209 most likely started with debromination, and resulted in the generation of lower PBDEs congeners, which was then easier to be broken down via ring opening. In this study, lower PBDEs congeners were detected but only in very low amount. It may due to the intracellular enzymatic degradation of BDE 209 which happens in the bacterial cell (Tang et al. 2016). Furthermore, other degradation mechanisms are likely to be involved in the biodegradation process, such as the hydroxylation.

Hydroxylation reaction is an important step in the degradation of halo aromatic compounds by organisms (Wang et al. 2016). It was revealed that PBDEs could be transformed to hydroxylated metabolites in many organisms, such as fish, plants, rats, mice, and humans (Kim et al. 2014; Shi et al. 2015). In this study, the deducted OH-Octa-BDEs, OH-Hexa-PBDEs and OH-Tri-PBDEs suggested BDE 209 could be degraded into OH-PBDEs through hydroxylation. This finding is consistent with a former research that *Burkholderia xenovorans* LB400 convert most of a mono-BDE to a hydroxylated mono-BDE (Robrock et al. 2009). Besides, Cao et al. found BDE 47 was more easily to transform via hydroxylation by *Phanerochaete chrysosporium* (Cao et al. 2017). The *m/z* 163 was proposed to be bromo-pentanal indicated ring opening process may occurred in the BDE 209 aerobic degradation by strain WZN-1. Previously, Xu and Wang found *Phlebia lindtner*i JN45 could degrade BDE 209 through debromination, hydroxylation, and ring-opening reactions (Xu and Wang 2014). BDE 209 degradation by *Bacillus pumilus* LY2 were supposed to be three possible metabolic pathways: debromination, hydroxylation, and cleavage of the diphenyl ether bond (Wang et al. 2016). BDE 209 has a symmetric molecular structure and good chemical stability. Therefore, the biodegradation of BDE 209 most likely started with debromination and hydroxylation, and resulted in the generation of lower PBDEs congeners or OH-PBDEs, which was then easier to be broken down via ring opening. The present study indicated that BDE 209 was successively biodegraded to BDE 28 via a debromination process, and the proposed OH-PBDEs and bromo-pentanal supported the possible of hydroxylation and ring opening process. The results indicated further mineralization of in the tricarboxylic acid cycle (TCA) could be possible. Further researches of BDE 209 degradation by strain WZN-1 requires using standard substances verify these possible hydroxylation and ring opening products.

So far, very little is known about aerobic microbial transformation of PBDEs by microorganisms and the enzymes involved in PBDEs transformation (Robrock et al. 2011). In the present study, the significant upregulation of the four BDE 209 degradation genes confirmed the debromination, hydroxylation and ring opening process of BDE 209 degradation by strain WZN-1. The functional annotation of the genome showed that the hydrolase gene is an alpha/beta hydrolase in NR database. The KEGG database suggested this gene is dhaA haloalkane dehalogenase, and has xenobiotics biodegradation and metabolism function. The dioxygenase gene in strain WZN-1 was identified as an aromatic ring-opening dioxygenase via COG database, which suggested the potential ring opening ability of strain WZN-1. Moreover, the dehalogenase gene 1 and dehalogenase gene 2 were identified as haloacid dehalogenases of *Stenotrophomonas maltophilia* from the NR database. Previous study showed that enzymes were involved in the PCB degradation, transport processes, energetic metabolism, electron transport, and carbon metabolism(Zhang et al. 2014). Dioxygenase enzymes are responsible for degradation of diphenyl ether and may be responsible for degradation of less brominated PBDEs (Lee et al. 2011). Additionally, the 2D electrophoresis (2-DE) and matrix-assisted laser desorption/ionization time of flight mass spectrometry (MALDI-TOF MS) were used to identify upregulated proteins during PCB degradation, genes encoding for dioxygenase, ABC transporters, transmembrane proteins, electron transporter, and energetic metabolism proteins were significantly upregulated. Similarly, Tang et al. also suggested that enzymes and genes might act as functional protein in BDE 209 biodegradation, ATP synthase and ABC transporter permease played a critical role in BDE 209 transport from extracellular environment to intracellular cells (Tang et al. 2016). Besides the four identified BDE 209 degradation genes, we also found twelve of ATP synthases, thirty-six of ABC transporter permeases, nineteen of hydroxylases, and sixty-five of oxygenase in strain WZN-1 (data not shown), which indicates that there may more genes involved in the biodegradation of BDE 209 by strain WZN-1.

In summary, a newly isolated bacterium *Stenotrophomonas* sp. strain WZN-1 could degrade 55.15% of 65 μg/L BDE 209 in 30 days under the optimized condition (i.e. pH 7, 25 °C, 0.5% salinity, 150 mL MSM volume). The degradation kinetic was fitted to a first-order kinetics model. The identified lower PBDEs congeners, OH-PBDEs and bromo-pentanal of the degradation of BDE 209 by WZN-1 suggested the degradation pathways included the debromination, and proposed hydroxylation and ring opening processes. Four identified genes was significantly induced and up-regulated by BDE 209, which indicated that these genes are of great importance in the BDE 209 degradation process. These findings could provide useful information for PBDE remediation applications and contribute to a better understanding of BDE 209 biodegradation process.

## Additional file

**Additional file 1.** Additional figures and tables.

## Abbreviations

BDE 209: decabromodiphenyl ether
PBDEs: polybrominated diphenyl ethers
POPs: persistent organic pollutants
THF: tetrahydrofuran
MSM: minimal salt medium
BMM: basal mineral medium
LB: Luria–Bertani
SEM: scanning electron microscopy
FCM: flow cytometry
NCI: negative chemical ionization
ESI: negative electrospray ionization mode
