# Supplementary material for: Biodegradation of decabromodiphenyl ether (BDE 209) by a newly isolated bacterium from an e-waste recycling area
Source: AMB Express. 2018 Feb 24;8:27. doi: 10.1186/s13568-018-0560-0 (PMC6890894; doi:10.1186/s13568-018-0560-0)
Supplement: Supplementary file 1 — Additional file 1. Additional figures and tables. [file 13568_2018_560_MOESM1_ESM.doc]

Journal: AMB Express

**Biodegradation of decabromodiphenyl ether (BDE 209) by a newly isolated bacterium from an e-waste recycling area**

**Zhineng Wu^1^, Miaomiao Xie^1^, Yao Li^1^, Guanghai Gao^1^, Mark Bartlam^2,3^****, Yingying Wang^1^**^*^

^1^Key Laboratory of Pollution Processes and Environmental Criteria (Ministry of Education), Tianjin Key Laboratory of Environmental Remediation and Pollution Control, College of Environmental Science and Engineering, Nankai University, Tianjin 300350, China

^2^State Key Laboratory of Medicinal Chemical Biology, Nankai University, Tianjin 300350, China

^3^College of Life Sciences, Nankai University, Tianjin 300071, China

*Corresponding author: Yingying Wang

E-mail address: [wangyy@nankai.edu.cn](mailto:wangyy@nankai.edu.cn)

Tel/Fax: +86 22 66229721

**Supplementary Information:**

**Table S1** Levels of factors tested in the orthogonal experiment.

**Table S2** Orthogonal experiment results.

**Table S3** Results of physiological-biochemical characteristics of strain WZN-1.

**Table S4** [Variance](javascript:void(0);) [analysis](javascript:void(0);) of orthogonal experiment results.

**Fig. S1.** (a) and (b) SEM of strain WZN-1 in LB culture medium after 30 h (the cells included in red circles are small cells of strain WZN-1). (c) FCM of strain WZN-1 grown in MSM with 65 μg/L of BDE 209 for 15 d (FL1: Green fluorescence; FL3: red fluorescence).

**Fig. S2.** The neighbourhood trees based on the genome of strain WZN-1. (a) neighbourhood tree of the full length of 16S rRNA sequence of strain WZN-1. (b) neighbourhood tree of the gene family based on the genome of strain WZN-1.

**Fig. S3.** Maximum biomass of strain WZN-1 in different LB concentrations in 36 h.

**Fig. S4.** Biodegradation kinetics of BDE 209 by strain WZN-1 under the optimal condition.

**Fig. S5.** GC-MS chromatogram of BDE 209 degradation by strain WZN-1. (a) standard 14 PBDE congeners, (b) control after 30 d, (c) incubation for 15 d, (d) incubation for 30 d.

**Table S1** Levels of factors tested in the orthogonal experiment.

| Levels | pH | Temperature (°C) | Salinity (%) | Volume (mL) | BDE 209 Concentration (μg/L) |
| --- | --- | --- | --- | --- | --- |
| 1 | 5 | 20 | 0 | 50 | 50 |
| 2 | 7 | 25 | 0.5 | 100 | 100 |
| 3 | 8 | 30 | 1 | 150 | 250 |
| 4 | 9 | 35 | 2 | 200 | 500 |
| 5 | 11 | 40 | 4 | 250 | 750 |

**Table S2** Orthogonal experiment results.

| Run | Factors and levels | | | | | BDE 209 degradation (%) |
| --- | --- | --- | --- | --- | --- | --- |
|  | pH | Temperature  (°C) | Salinity  (%) | Volume  (mL) | BDE 209  (μg/L) |  |
| 1  2  3  4  5  6  7  8  9  10  11  12  13  14  15  16  17  18  19  20  21  22  23  24  25  Mean 1  Mean 2  Mean 3  Mean 4  Mean 5  Range | 5  5  5  5  5  7  7  7  7  7  8  8  8  8  8  9  9  9  9  9  11  11  11  11  11  57.08  25.92  13.84  8.68  7.66  49.41 | 20  25  30  35  40  20  25  30  35  40  20  25  30  35  40  20  25  30  35  40  20  25  30  35  40  25.80  28.15  20.67  20.72  17.83  10.33 | 0  0.5  1  2  4  0.5  1  2  4  0  1  2  4  0  0.5  2  4  0  0.5  1  4  0  0.5  1  2  16.46  29.47  26.11  25.16  15.97  13.49 | 50  100  150  200  250  150  200  250  50  100  250  50  100  150  200  100  150  200  250  50  200  250  50  100  150  19.45  21.18  31.18  25.13  16.14  15.13 | 50  100  250  500  750  500  750  50  100  250  100  250  500  750  50  750  50  100  250  500  250  500  750  50  100  19.30  20.91  23.50  25.81  23.65  6.51 | | 46.46 ± 1.57 | | --- | | 74.04 ± 0.67 | | 65.37 ± 3.23 | | 57.11 ± 2.15 | | 42.41 ± 0.98 | | 50.00 ± 1.44 | | 37.33 ± 1.68 | | 24.44 ± 0.79 | | 9.70 ± 2.01 | | 8.11 ± 1.57 | | 6.03 ± 3.24 | | 21.84 ± 2.37 | | 5.84 ± 0.79 | | 23.20 ± 1.63 | | 12.29 ± 0.91 | | 11.18 ± 2.64 | | 6.59 ± 2.10 | | 3.59 ± 1.28 | | 6.88 ± 2.64 | | 15.13 ± 0.69 | | 15.32 ± 3.27 | | 0.96 ± 2.50 | | 4.12 ± 1.93 | | 6.70 ± 2.33 | | 11.20 ± 3.09 | |  | |

Note: The degradation time was 7 d. “Mean 1, mean 2, mean 3, mean 4 and mean 5” refers to mean BDE 209 degradation % values of five factors at five different levels (e.g. Mean 1 for pH was the average BDE 209 degradation % at pH 5); “Range” refers to the range of those mean values.

**Table S3 Results of** **physiological-biochemical characteristics of strain WZN-1.**

| Biochemical and culture conditions | Results |
| --- | --- |
| Gram staining | - |
| Glucose utilization | - |
| V-P test | - |
| Catalase | + |
| Oxidase | - |
| Nitrate reduction | - |
| Flagella staining | + |
| Citrate utilization | + |
| Hydrolysis of starch | - |
| M.R. test | - |
| Urea test | - |
| Gelatin liquefaction | + |

“+” means positive reaction, “-” means negative reaction.

**Table S4** [Variance](javascript:void(0);) [analysis](javascript:void(0);) of orthogonal experiment results.

| Sources | df | SS | MS | *F* value | *P* Level |
| --- | --- | --- | --- | --- | --- |
| pH | 4 | 8468.00 | 2117.00 | 14.92 | 0.01 |
| Temperature (°C)  Salinity (%) | 4  4 | 355.40  737.90 | 88.85  184.47 | 0.63  1.30 | 0.67  0.40 |
| Volume (mL) | 4 | 676.10 | 169.03 | 1.19 | 0.44 |
| BDE 209 Concentration (μg/L) | 4 | 129.80 | 32.46 | 0.23 | 0.91 |
| Error  Total | 4  24 | 567.60  10934.8 | 141.90 |  |  |

Note: df refers to degrees of freedom; SS refers to sum of sequences; MS refers to mean square.

*P* level less than 0.05 indicates the model terms are significant.


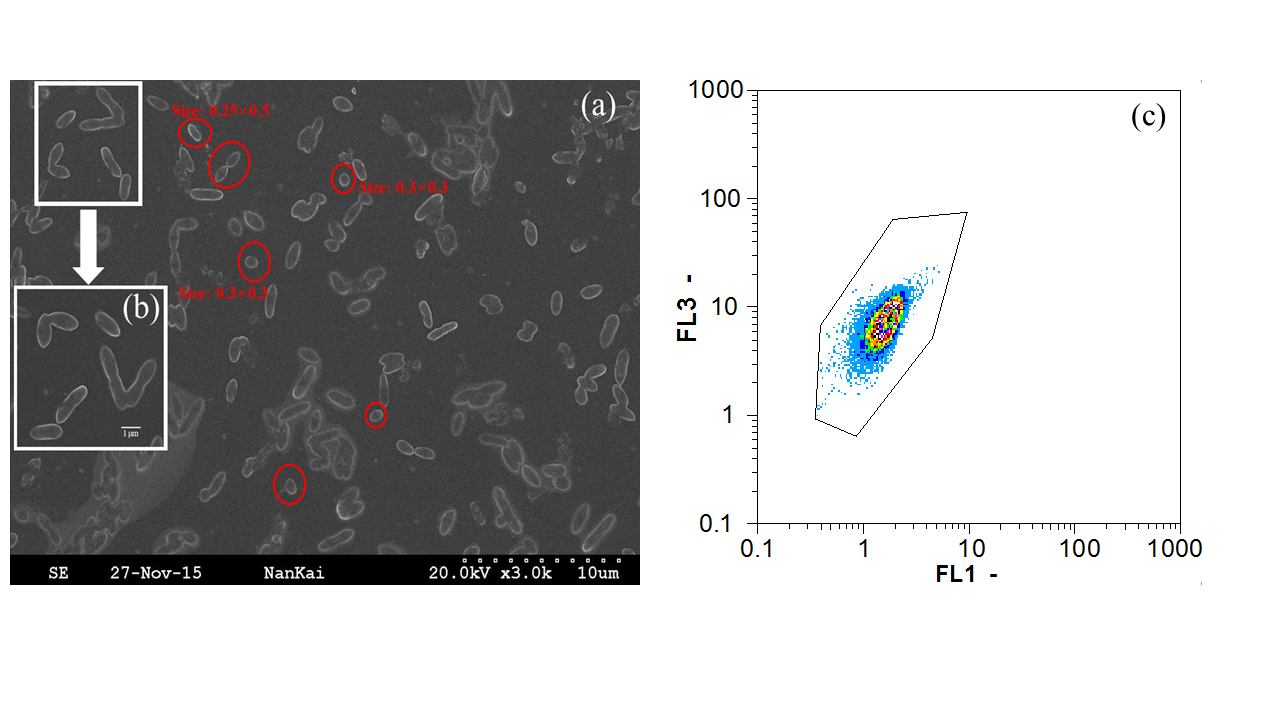


**Fig. S1.** (a) and (b) SEM of strain WZN-1 in LB culture medium after 30 h (the cells included in red circles are small cells of strain WZN-1). (c) FCM of strain WZN-1 grown in MSM with 65 μg/L of BDE 209 for 15 d (FL1: Green fluorescence; FL3: red fluorescence).


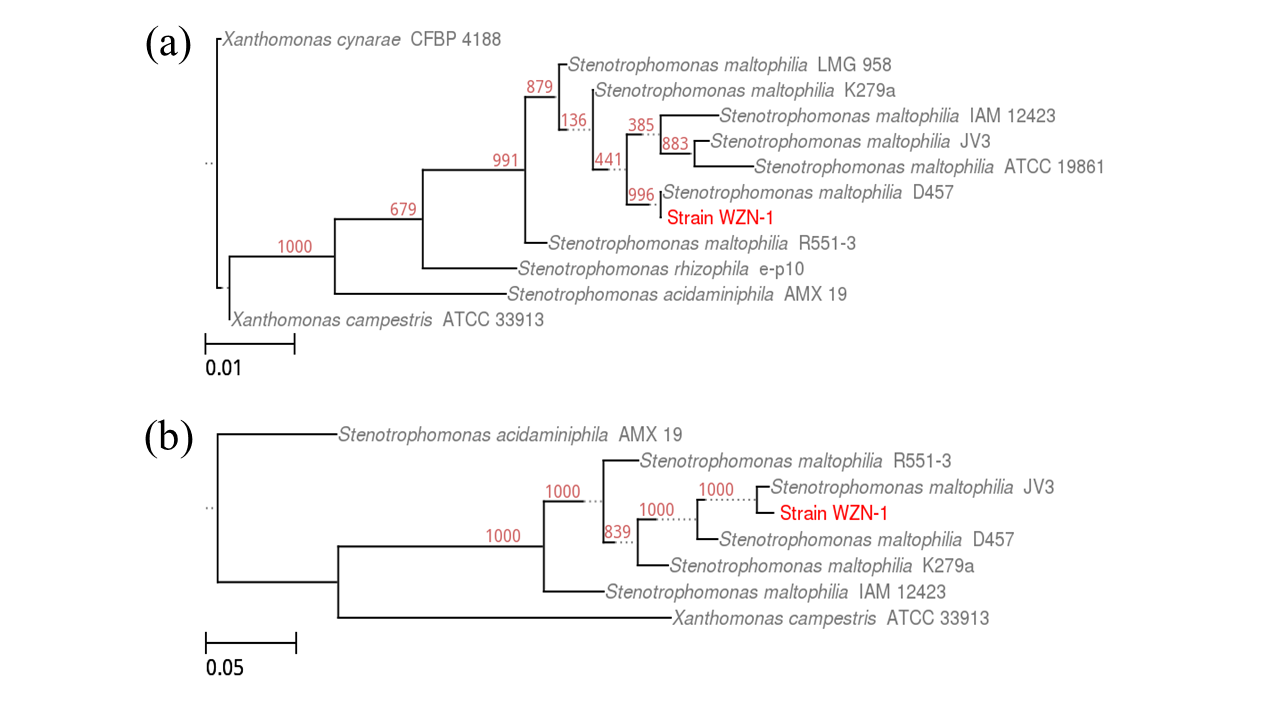


**Fig. S2.** The neighbourhood trees based on the genome of strain WZN-1. (a) neighbourhood tree of the full length of 16S rRNA sequence of strain WZN-1. (b) neighbourhood tree of the gene family based on the genome of strain WZN-1.


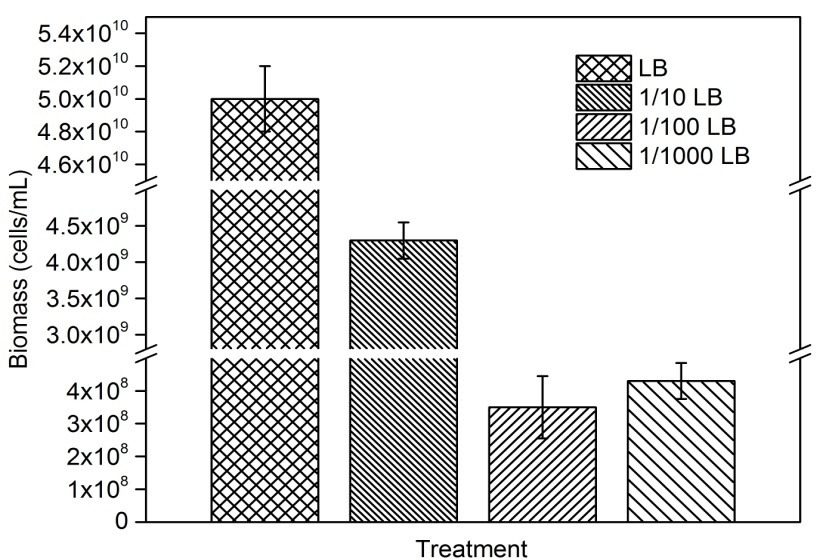


**Fig. S3.** Maximum biomass of strain WZN-1 in different LB concentrations in 36 h.


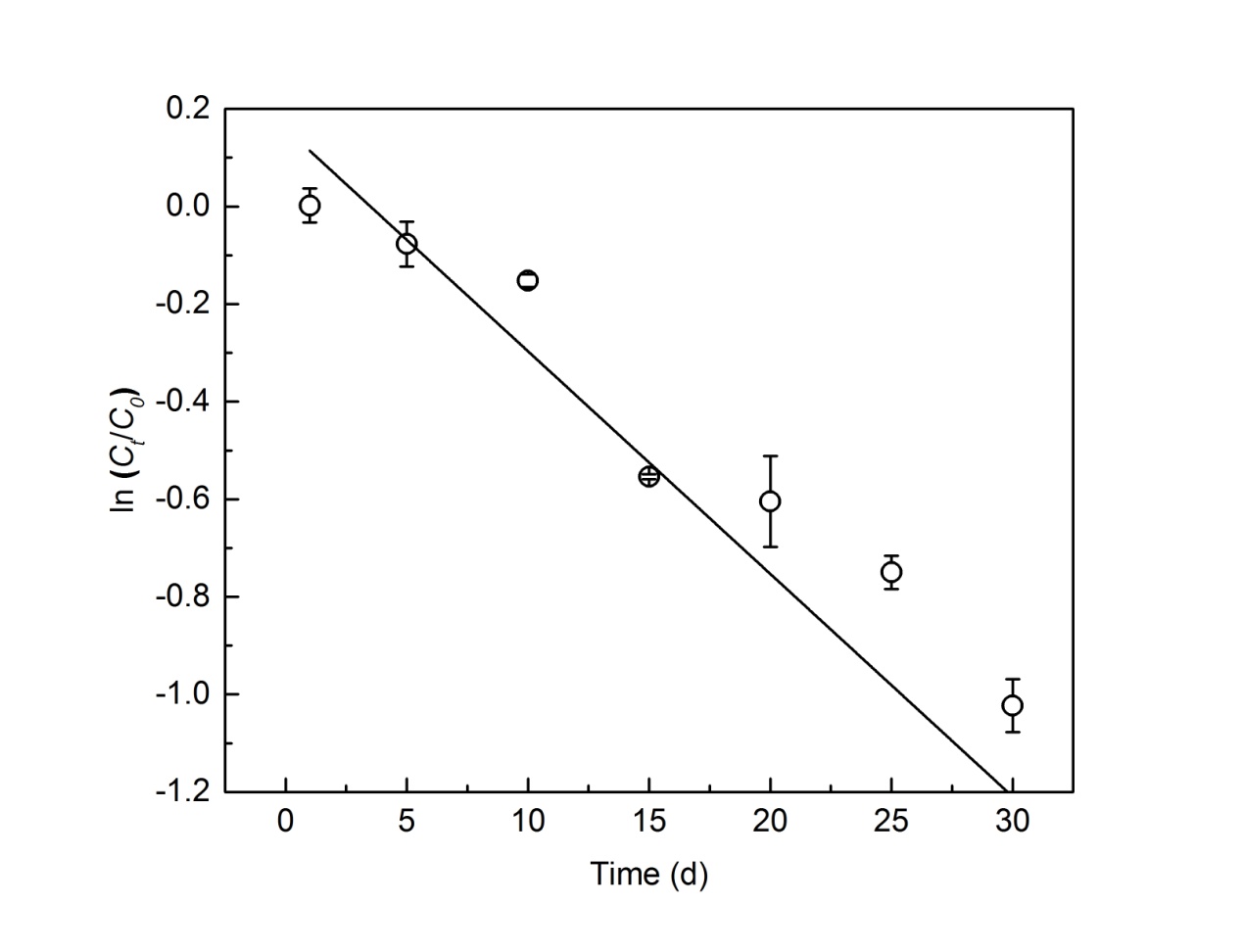


**Fig. S4.** Biodegradation kinetics of BDE 209 by strain WZN-1 under the optimal condition.

**
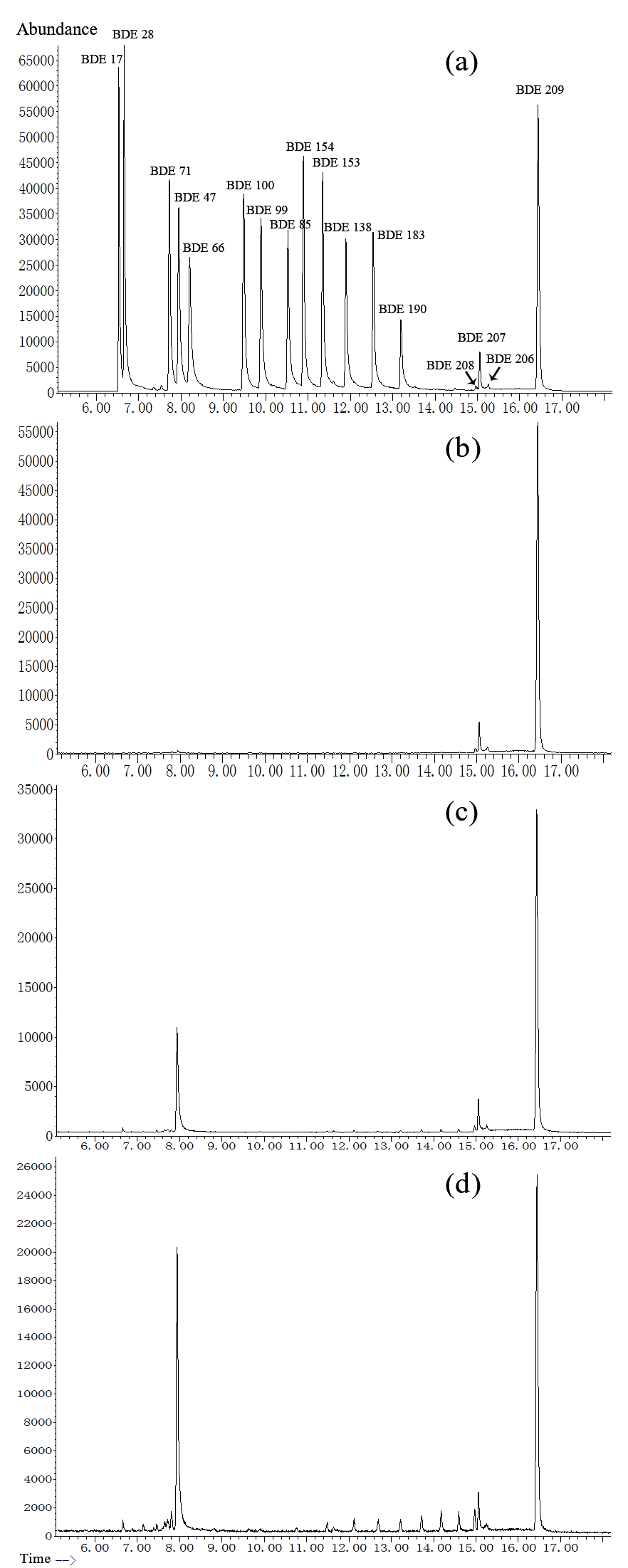
**

**Fig. S5.** GC-MS chromatogram of BDE 209 degradation by strain WZN-1. (a) standard 14 PBDE congeners, (b) control after 30 d, (c) incubation for 15 d, (d) incubation for 30 d.
